# Epithelial defects after penetrating keratoplasty in infectious keratitis: An analysis of characteristics and risk factors

**DOI:** 10.1371/journal.pone.0208163

**Published:** 2018-11-28

**Authors:** Shanshan Wan, Jun Cheng, Yanling Dong, Lixin Xie

**Affiliations:** 1 Department of Ophthalmology, Renmin Hospital of Wuhan University, Wuhan, Hubei Province, China; 2 State Key Laboratory Cultivation Base, Shandong Provincial Key Laboratory of Ophthalmology, Shandong Eye Institute, Shandong Academy of Medical Sciences, Qingdao, Shandong Province, China; Universidad de Monterrey Division de Ciencias de la Salud, MEXICO

## Abstract

To investigate the clinical characteristics, treatment, risk factors of occurrence and graft transparency of corneal epithelial defects after penetrating keratoplasty in patients with infectious keratitis. 594 patients (594 eyes) with infectious keratitis treated by penetrating keratoplasty at Shandong Eye Institute were reviewed retrospectively between January 2008 and January 2018. We investigated the demographic data, diameter and sources of graft, onset time, location, scope, time of healing and treatment of epithelial defects, as well as other postoperative complications and graft clarity. 114 of the 594 grafts (19.2%) that developed epithelial defects were included in the epithelial defects group, while the other 480 patients were classified in the non-defect group. The mean age of patients with epithelial defects was statistically greater than that of patients without epithelial defects (P = 0.006). The epithelial defects group accounted for a larger proportion of male patients (P<0.001). The proportion of patients with a graft diameter >9mm in the epithelial defect group (29.8%) was more than that in the non-defects group (16.3%) (P = 0.001). The incidence of epithelial defects significantly differed among the pathogenic causes of infectious keratitis (P = 0.002). The incidence of graft infection (21.1%, 9.2%, respectively, P<0.001) and graft dysfunction (7.9%, 2.5%, respectively, P = 0.012) in the epithelial defect group was higher than in the non-defects group. Multivariate logistic regression revealed that male sex (P = 0.001), age ≥ 60 years (P = 0.024), graft diameter >9mm (P = 0.001), bacterial (P = 0.039) and herpes simplex keratitis (P = 0.008), rheumatism (P = 0.031) and cancer treated with chemo- or radiotherapy (P = 0.032) were independent risk factors for epithelial defects. Graft clarity after epithelial defects were significantly differed between fungal and viral infections (P<0.001). We found that being an elderly male patient, a graft diameter >9 mm, bacterial and viral keratitis and systemic diseases (including rheumatism and cancer treated with chemo- or radiotherapy) were independent risk factors for postoperative epithelial defects.

## Introduction

Corneal epithelial defects are common after penetrating keratoplasty (PK). Delayed epithelial healing can lead to secondary infection, angiogenesis, corneal ulcer formation, lower postoperative vision and even an ultimate failure of corneal transplantation. At present, specific mechanisms of epithelial healing remain unclear. Studies have reported higher incidences of epithelial defects after herpes simplex [1, 2] or adenoviral [3] keratitis when compared to those of patients without viral infection. This phenomenon is considered to be related to a reduction of corneal sensitivity as well as a proliferation and differentiation of epithelial cells. However, there are no studies evaluating how bacterial, acanthamoeba and fungal infections affect epithelial defects. This study is a retrospective analysis of epithelial defects after PK in the setting of infectious keratitis caused by various pathogens. We also explore associated risk factors so as to guide clinical treatment and improve graft survival.

## Materials and methods

### Patients

This retrospective study was approved by the institutional review board of Shandong Eye Institute. All research procedures conformed to the Helsinki Declaration, informed consent was waived because of the retrospective nature of the study and the data were analyzed anonymously. Cases of patients diagnosed with infectious keratitis who underwent PK between January 2008 and 2018 were reviewed.

### Inclusion and exclusion criteria

Patients diagnosed with bacterial, fungal, acanthamoeba or herpes simplex keratitis, according to diagnostic criteria consistent with those in existing literature, were included in this study [4]. Patients underwent PK if they were not effectively managed with medications or suffered deep infiltration of the inner corneal matrix or corneal perforation. All patients were followed up for more than 6 months. Patients who suffered exposure keratitis, immune corneal ulceration, Terrien’s marginal degeneration, or corneal dystrophy, as well as those who underwent ocular surface reconstruction, were excluded. In addition, patients who suffered infection in the setting of any of the aforementioned conditions, were infected by two or more pathogens, underwent keratoplasty in addition to other surgery, underwent corneal transplantation prior to PK or were lost to follow up were excluded.

Patients who suffered delayed epithelial healing (defined as lasting for over 6 days after PK) or epithelial defects after epithelialization were included in the epithelial defects group [1]. Patients with epithelialization within 6 days after surgery were included in the non-defect group. Patients with epithelial defects underwent conjunctival scraping and confocal microscopy examination in order to rule out a possibility of infection. Corneal epithelial defects were visualized with fluorescein staining under slit-lamp microscopy. Extent of defects was recorded as a maximum on the vertical axis. Epithelium was defined as healed when there was complete epithelialization and no fluorescein staining. Healing time was recorded from the time of defect detection to the time of healing. Epithelial defects within a diameter of 3mm from the corneal center were classified as central defects. Defects within a diameter of 3mm to 6mm from the corneal center were classified as mid-peripheral defects. Defects at least 6mm in diameter away from the corneal center were classified as peripheral defects. At the end of follow-up, grafts that were not clear in the central visual axis were classified as opaque [5].

### Perioperative management of penetrating keratoplasty

Routine preoperative evaluation included collection of patient medical history as well as slit lamp microscopy, confocal microscopy, corneal scraping and etiology cultured. All operations were performed by the same surgeon. All donor cornea were stored in DX solution, as previously described[6], or Optisol GS (Bausch & Lomb, Rochester, NY) preserving solution; their states were consistent according to unified quantitative grading by the Shandong Eye Institute eye bank. Routine PK was performed using a Hessburg-Barron trephine with a diameter of 0.25mm or 0.5mm larger than the graft bed. The average diameter of donor button was 8.5±1.1mm. Grafts were sutured with interrupted 10–0 nylon sutures. A balanced saline solution was continuously applied to prevent corneal epithelial drying. No curettage was performed on corneal epithelium during surgery.

Patients were routinely monitored daily for 5 to 7 days postoperatively while in hospital. Patients were then monitored weekly for 1 month postoperatively as outpatients and subsequently followed-up during monthly visits over the next half year. Afterwards, follow-up visits were scheduled once every 2–3 months when the graft was deemed stable. Patients were administered orally prednisone (1mg/kg•d) daily postoperatively and dosage was subsequently tapered after 1 week, except patients with fungal keratitis. Patients diagnosed with bacterial keratitis were treated with appropriate antibiotics according to antimicrobial susceptibility; drugs were discontinued within 2 weeks. Patients diagnosed with herpes simplex keratitis were treated with oral acyclovir (3 x 400mg) daily for 3 months. In addition, these patients were treated with topical ganciclovir eye drops 4 times per day and ganciclovir eye gel once per night for 6 months. Patients diagnosed with fungal keratitis were treated with 5% natamycin eye drops; antibiotic eye drops were administered locally. Patients diagnosed with acanthamoeba keratitis were treated with 0.02% chlorhexidine and 0.5% metronidazole eye drops after surgery, 4–6 times a day, for at least 1 month. Patients suffering bacterial or viral keratitis were treated with 1% prednisolone acetate eye drops within 3 weeks after PK, later changed to 0.02% fluorometholone for 6 months. Glucocorticoid administration was terminated in patients suffering fungal or acanthamoeba keratitis 2 weeks after corneal transplantation. If primary disease did not recur within 2 weeks after PK, glucocorticoids could be used locally in an exploratory fashion with close patient monitoring[7]. An immunosuppressant such as cyclosporine A or tacrolimus was applied locally 4 times per day for the first 3 months after operation; afterwards the medication was gradually tapered over the course of the next 6 months and applied twice daily.

### Statistical data

General demographic information (such as age, gender, occupation, and history of systemic disease), diameter and source of graft, pertinent time points, location, scope, healing time and treatment of epithelial defects were analyzed. Postoperative complications (such as graft rejection, infection, and functional decompensation) and other graft outcomes, such as repeat PK or evisceration, were extracted from medical records (S1 Dataset). Data pertaining to graft clarity and opacity during the follow-up period were also reviewed.

### Statistical analysis

All data were analyzed with SPSS software version 22.0. Clinical characteristics of patients with or without epithelial defects were compared using Chi-squared, Mann–Whitney U or Fisher’s exact tests. The Kruskal–Wallis test was performed to compare the range, healing area and duration of epithelial defects due to infectious keratitis of different etiologies. Logistic regression analyses were used to evaluate risk factors associated with epithelial defects, and results were represented by odds ratios (95% confidence intervals). P<0.05 was considered as statistically significant.

## Results

### Demographic and clinical characteristics

Of patients that underwent PK at Shandong Eye Institute from January 2008 to January 2018, 480 eyes were included in the non-defect group and 114 eyes in the epithelial defect group. There were 314 males (65.4%) in the non-defect group and 96 males (84.2%) in the epithelial defect group (P<0.001). Mean ages were 51.3±14.0 years and 55.4±12.1 years (P = 0.006) for the two groups, respectively. Results revealed that patients in the epithelial defect group were older and more likely to be male than those in the non-defect group. We found no statistical difference between the two groups in terms of outdoor occupations (P = 0.60)(Table 1). Mean follow-up times of two groups without and with epithelial defects were 12.1±13.4 and 11.3±12.9 months, respectively (P = 0.689).

**Table 1:** Demographic and clinical characteristics of patients with epithelial defects after penetrating keratoplasty for infectious keratitis.

Clinical Factors	With epithelial defects (n = 114)	Without epithelial defects (n = 480)	P value*
Sex No(%)			<0.001
Male	96(84.2)	314(65.4)	
Female	18(15.8)	166(34.6)	
Age(mean±SD)years	55.4±12.1	51.3±14.0	0.006^※^
Eye			0.49
Right	63(55.3)	248(51.7)	
Left	51(44.7)	232(48.3)	
Occupation			0.60
Indoors	44(38.6)	198(41.3)	
Outdoors	70(61.4)	282(58.7)	
Diameter of graft>9mm			0.001
Yes	34(29.8)	78(16.3)	
No	80(80.2)	402(83.7)	
Source of graft			0.67
DX	99(86.8)	422(87.9)	
Optisol	15(13.2)	56(12.1)	
Complications and outcomes			
Graft rejection	13(11.4)	112(23.3)	0.005
Graft infection	26(22.8)	44(9.2)	<0.001
Graft dysfunction	9(7.9)	12(2.5)	0.012^§^
Repeat PK	7(6.1)	20(4.2)	0.36
Evisceration	3(2.6)	6(1.3)	0.51§
Systemic diseases			
Diabetes mellitus	7(6.1)	30(6.3)	0.97
Rheumatism	6(5.3)	8(1.7)	0.035#
Cancer patients treated with chemo- or radiotherapy	4(3.5)	4(0.8)	0.048#

*Chi-squared test.

^§^Chi-squared test with continuity correction.

^※^Mann–Whitney U test.

^#^Fisher’s exact test.

**Fig 1:**
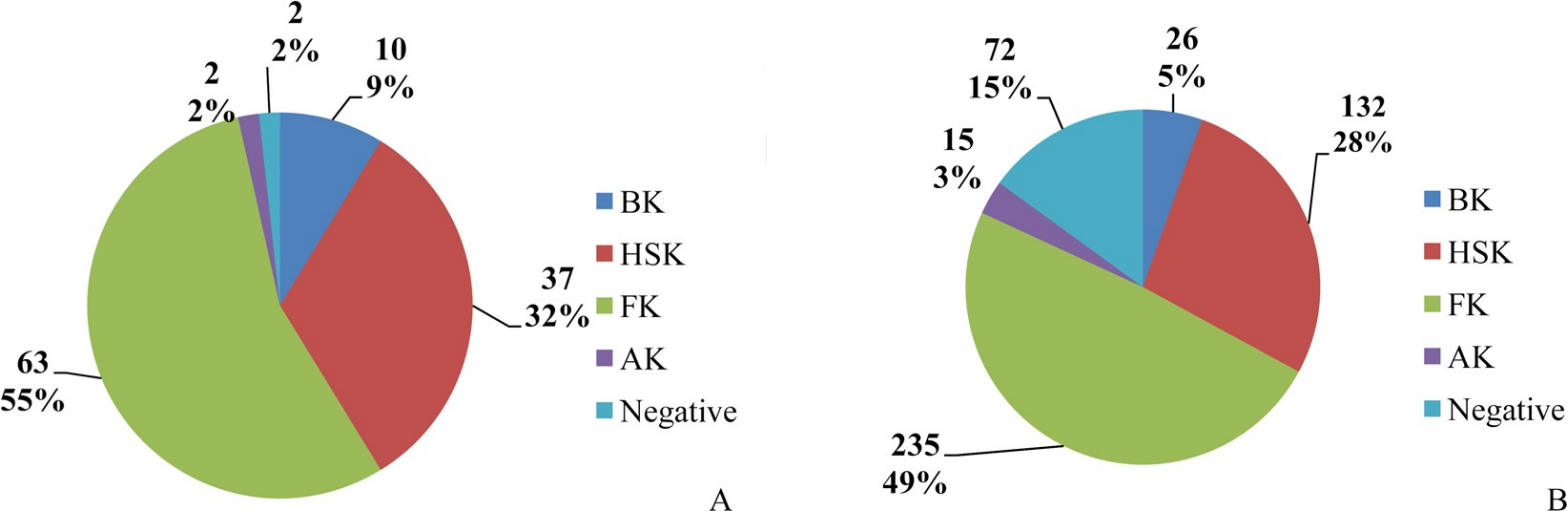
Indications for penetrating keratoplasty with and without epithelial defects. (A) Causes of epithelial defects after penetrating keratoplasty in 114 eyes from January 2008 to January 2018. (B) Indications for penetrating keratoplasty in 480 eyes without epithelial defects from January 2008 to January 2018.

In the epithelial defect group, percentages of patients who suffered fungal, bacterial, viral and acanthamoeba keratitis were 55.3% (63/114), 8.8% (10/114), 32.5% (37/114), and 1.8% (2/114), respectively. Causative pathogens remained unclear in two cases (1.8%). In the non-defect group, there were 235 cases of fungal (49%), 26 cases of bacterial (5.4%), 132 cases of viral (27.5%), and 15 cases of acanthamoeba (3.1%) keratitis. Causative pathogens remained unclear in 72 cases (15%). Causative pathogens differed significantly between the two groups (P<0.001) (Fig 1). There was no statistical difference between the two groups in terms of graft sources (P = 0.67). A greater percentage of patients in the epithelial defect group had graft diameters of over 9mm when compared to the non-defect group (34 patients; 29.8%, and 78 patients; 16.3%, respectively) (P = 0.001). There were 7 cases (6.1%) of diabetes in the epithelial defect group and 30 cases (6.3%) in the non-defect group (P = 0.97). There were 6 (5.3%) and 8 (1.7%) cases of rheumatoid-related immune diseases in the epithelial defect and non-defect groups, respectively (P = 0.035), and 4 cases of cancer after radiotherapy and chemotherapy in each group (3.5% and 0.8%, respectively) (P = 0.048).

### Characteristics of epithelial defects after PK

We found that epithelial defects occurred from 2 weeks to 48 months after PK. Defects were noted in 44 cases (38.6%) within 6 days to 2 weeks after surgery and in 88 cases (77.2%) after 2 weeks, among which 22 patients (19.3%) suffered recurrent epithelial defects. Epithelial defects were mainly localized to the central cornea (84 cases; 73.7%), mid-peripheral zone (12 cases; 10.5%), and peripheral zone (18 cases; 15.8%). The average size of epithelial defects was 12.0±13.1 mm^2^. Incidences of epithelial defects in bacterial, viral, fungal, and acanthamoeba keratitis were 27.8%, 21.9%, 21.1%, and 11.8%, respectively. Manifestations of epithelial defects in the setting of various causative pathogens statistically differed (P = 0.002). Data revealed that epithelial defects in bacterial keratitis were primarily noted two weeks after PK; defects in the setting of viral, fungal and acanthamoeba keratitis were found after 2 weeks at 73%, 54% and 100%, respectively (P = 0.045). Table 2 shows that the mean area of epithelial defects in viral and fungal keratitis was 10.4±11.0mm^2^ and 11.6±12.7mm^2^, respectively, larger than that of bacterial keratitis (7.6±6.5mm^2^).

**Table 2:** Characters of corneal defects after penetrating keratoplasty for different pathogenic causes of infectious keratitis.

		Bacterial (n = 36)	Viral (n = 169)	Fungal (n = 298)	Acanthamoeba (n = 17)	P value
Incidence of epithelial defects		10(27.8%)	37(21.9%)	63(21.1%)	2(11.8%)	0.002*
Number of healed		9(90%)	30(81.1%)	46(73%)	2(100%)	0.739^#^
Time at diagnosis of epithelial defects	≤2weeks	7(70%)	10(27%)	29(46%)	0	0.045^#^
	>2weeks	3(30%)	27(73%)	34(54%)	2(100%)	
Mean size of epithelial defects (mm^2^)		7.6±6.5	10.4±11.0	11.6±12.7	36.5±50.2	0.006^&^
Mean duration of epithelial healing (day)		13.3±6.7	10.8±6.0	10.6±5.6	8.5±2.1	0.232^&^

* Chi-squared test.

^#^ Fisher’s exact test.

^&^ Kruskal–Wallis test.

### Healing and treatment of epithelial defects

During the follow-up period, epithelial defects in 89 (78.1%) patients healed within 4–32 days after medical or surgical intervention. Graft ulcers formed in 17 patients (14.9%), 3 patients (2.6%) suffered evisceration, and 2 patients (1.8%) underwent repeat PK and suffered graft dysfunction, respectively. There was one case (0.9%) of endophthalmitis. Bacterial, viral, fungal, and acanthamoeba infections affected healing of epithelial defects in 9 (90%), 30 (81.1%), 46 (73%) and 2 (100%) eyes(Table 2), and the other two cases were pathogens undetected. No significant difference was found in the number of healing cases and duration between different subgroups (P = 0.739, 0.232).

Patients who suffered epithelial defects after PK were initially managed with simple medical therapy. If medical management was noted ineffective after 3 days, patients were subsequently treated with contact lenses. If medical management was ineffective and the condition was aggravating, surgical intervention was carried out. Simple pharmacotherapy (including artificial tears, autologous serum and glucocorticoid eye drops) successfully treated 8 (7%) patients while 5 cases (4.4%) were managed with contact lenses. In addition, 6 patients (5.3%) were treated with amniotic membrane transplantation (AMT). Of these patients, two underwent AMT more than twice; 1 patient healed successfully while the others underwent tarsorrhaphy. A total of 85 patients (74.6%) underwent tarsorrhaphy, of which 24 underwent tarsorrhaphy twice or more. Time of tarsorrhaphy ranged from 5 days to 39 months postoperatively. Most of the patients (63; 74.1%) eventually healed. Sutures were adjusted in 9 patients (7.9%) while 1 case (0.9%) involved conjunctival flap covering combined with blood glucose control or rheumatoid related indicators, including administration of non-steroidal anti-inflammatory drugs and glucocorticoid following internist's advice.

### Graft transparency and other complications

**Fig 2:**
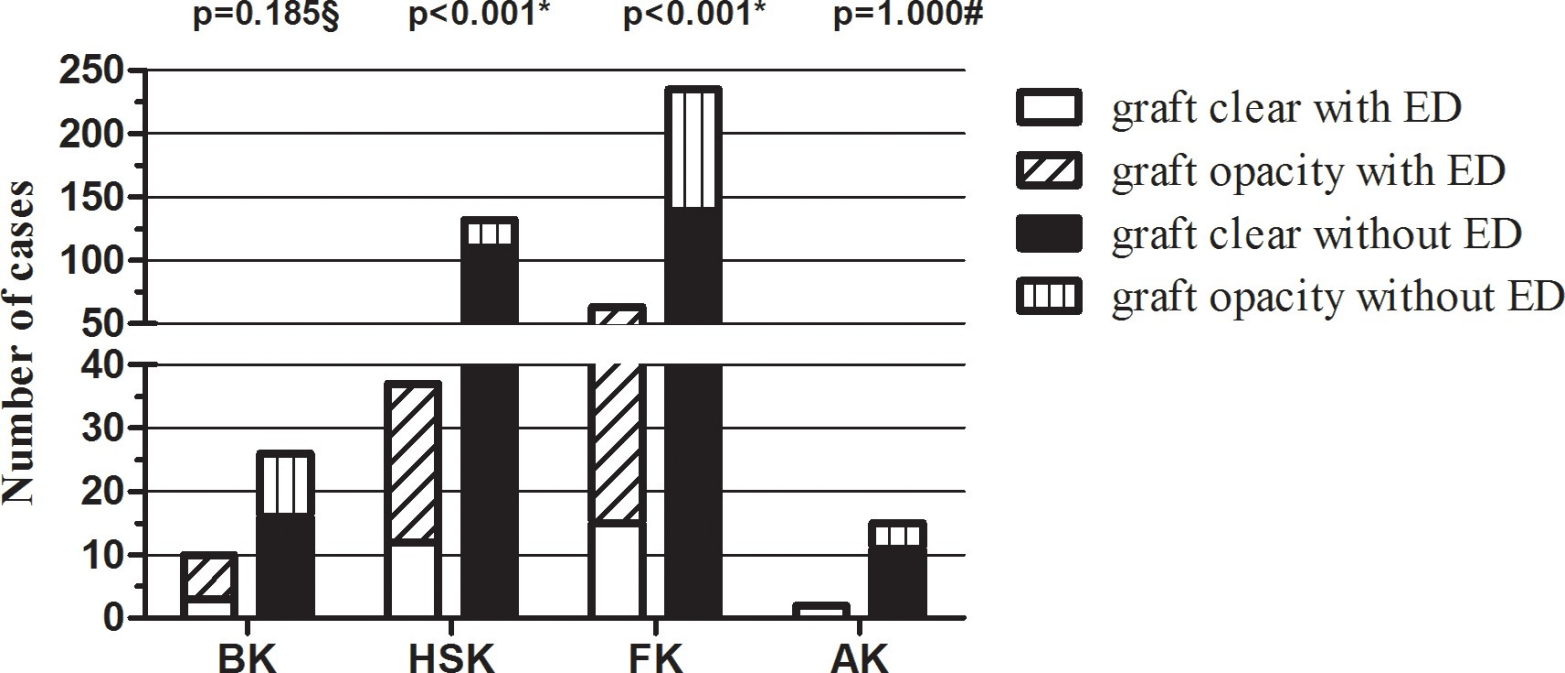
Comparison of graft clarity after penetrating keratoplasty among different etiologies of infectious keratitis in patients with and without epithelial defects during the follow-up period. *Chi-squared test. ^§^Chi-squared test with continuity correction. ^#^Fisher’s exact test.

Corneal graft transparency was observed in the setting of keratitis caused by different pathogens (Fig 2). In patients who suffered bacterial keratitis, there were 3 (30%) and 16 (61.5%) eyes with transparent grafts in groups with and without epithelial defects, respectively, in addition to 12 (32.4%) and 110 (83.3%) eyes in herpes simplex keratitis, 15 (23.8%) and 139 eyes (59.1%) in fungal keratitis, and 2 (100%) and 139 (59.1%) eyes in acanthamoeba keratitis, respectively. Graft clarity after epithelial defects was found to significantly differ between patients affected by fungal and viral keratitis (P<0.001, 0.004). Other postoperative complications (i.e. graft rejection) in groups with and without epithelial defects occurred in 13 (11.4%) and 112 (23.3%) patients, respectively (P = 0.005).

In the epithelial defect group, more patients (22.8%) suffered graft infection than did non-defect group patients (9.2%) (P<0.001). Furthermore, there were 9 (7.9%) and 12 cases (2.5%) of graft decompensation (P = 0.012) in epithelial defect and non-defect groups, respectively. Repeat PK was performed in 7 (6.1%) and 20 cases (4.2%) in the defect and non-defect groups, respectively. There were 3 (2.6%) and 6 cases (1.3%) of evisceration in defect and non-defect groups, respectively.

### Risk factors for developing epithelial defects

Univariate analysis revealed that gender (P<0.001), a graft diameter >9mm (P<0.001), bacterial (P = 0.017), viral (P = 0.038) and fungal (P = 0.041) infection, systemic diseases (including rheumatism (P = 0.031) and cancer treated with chemoradiotherapy (P = 0.040)) were risk factors for the development of epithelial defects after PK. Diabetes was not found to be a statistically significant risk factor (P = 0.301). Further multivariate logistic regression (Table 3) revealed that male sex (P = 0.001), age ≥ 60 years (P = 0.024), a graft diameter >9mm (P = 0.001), bacterial (P = 0.039) and viral (P = 0.008) keratitis, rheumatism (P = 0.031) and cancer treated with chemoradiotherapy (P = 0.032) were independent risk factors for the development of postoperative epithelial defects.

**Table 3:** Regression analysis of various risk factors for epithelial defects after penetrating keratoplasty.

	Univariate Analysis			Multivariate Analysis		
Risk Factor	OR	95%CI	P value	OR	95%CI	P value
Sex (male)	2.82	1.65–4.83	<0.001	2.59	1.49–4.52	0.001
Age≥60 years	1.51	0.97–2.34	0.065	1.71	1.07–2.73	0.024
Graft diameter>9mm	2.22	1.42–3.45	<0.001	2.41	1.46–3.98	0.001
Primary disease			0.019			0.014
Bacterial keratitis	2.39	1.17–4.91	0.017	2.19	1.04–4.60	0.039
Herpes simplex keratitis	1.75	1.03–2.95	0.038	2.13	1.22–3.70	0.008
Fungal keratitis	1.67	1.02–2.73	0.041	1.62	0.98–2.70	0.062
Acanthamoeba keratitis	0.83	0.09–1.93	0.766	0.75	0.21–2.67	0.652
Diabetes mellitus	0.98	0.42–2.29	0.965	1.02	0.42–2.47	0.975
Rheumatism	3.28	1.11–9.64	0.031	3.72	1.13–12.25	0.031
Cancer patients with chemo- or radiotherapy	4.33	1.07–17.57	0.040	5.33	1.15–24.67	0.032

## Discussion

PK remains the mainstream of therapy for infectious keratitis. Available literature on postoperative complications mainly focuses on graft rejection, high intraocular pressure and corneal ulceration; however, reports concerning epithelial defects, which directly affect the status of corneal grafts, are lacking. Early epithelial defects occur in 14%–100% of patients after PK and usually heal within 1 week postoperatively. However, 3%–7% of treated patients remain unhealed after 2 weeks, thus suffering persistent epithelial defects [8–10]. This study found 114 patients (19.2%) to have suffered epithelial defects after PK for infectious keratitis, a higher percentage than other studies reported.

We compared the clinical features and prognosis of infectious keratitis caused by different pathogens after PK in order to identify possible risk factors for epithelial defects in patients who suffer this condition. Demographic data revealed that patients who suffered epithelial defects were older than those who did not (P = 0.006). Feiz et al. [11] also reported corneal lesions as more likely to develop with increasing patient age. It is significantly more difficult for epithelial barrier function to recover as patient age increases [12]. Ocular immunity declines with age as well [13], likely affecting the process of epithelial healing. Furthermore, the majority of our patients in both groups were male, and the proportion of males in the epithelial defect group was higher than that in the non-defect group (84.2%, 65.4%, P< 0.001). Logistic regression analysis revealed that male sex was an independent risk factor for epithelial defects after PK, consistent with previous reports [14]. We also found that a graft diameter exceeding 9 mm was a susceptibility factor to epithelial defect formation after corneal surgery.

Generally, epithelial defects were mainly located in the middle of the graft, consistent with the tendency of epithelial cells to be located above the basement membrane as well as to proliferate and migrate centripetally to form integral epithelial structures [15]. Our analysis revealed that epithelial defects could occur from 2 weeks to 48 months after surgery, with 44 cases (38.6%) occurring within 6 days to 2 weeks after surgery, and 88 cases (77.2%) developing after 2 weeks, among which 22 (19.3%) suffered recurrent epithelial defects. Although the integrity of the epithelium plays a key role in corneal clarity, prior literature [8] reported the epithelial state on the first postoperative day to have no predictive value 3 months later. Among our studied patient population, viral and fungal keratitis first appeared mostly after 2 weeks; further follow-up of graft status revealed viral (P< 0.001) and fungal (P< 0.001) keratitis to significantly affect the degree of graft transparency after epithelial defects. Our findings suggest that the occurrence of primary disease and late epithelial defects influence graft status, however, larger randomized clinical trials are needed to further explore any possible associations.

Both systemic and local factors influenced the development of epithelial defects. Local factors included abnormal ocular surface structure and function, such as decreased blinking frequency, eyelid deficits, poor stability of tear film, a lack of limbal stem cells, and neurotrophic keratitis [16, 17]. Systemic factors that could interfere with re-epithelialization include Stevens–Johnson syndrome, cicatricial pemphigoid and diabetes [18]. We found systemic diseases (including rheumatism and cancer treated with chemo- or radiotherapy) to have been independent risk factors for epithelial defect formation, but diabetes not to have been statistically significant according to logistic analysis. We observed infectious keratitis complicated by pre-existing diabetes mellitus to heal more slowly, consistent with delayed wound healing and corneal nerve regeneration associated with diabetes. Although another retrospective study performed at our institute found that diabetic patients with fungal keratitis exhibited delayed re-epithelialization [19], we found no statistical significance between the groups. Sample size and the influence of other factors may have affected results.

Univariate analysis revealed that bacterial (P = 0.017), viral (P = 0.038) and fungal (P = 0.041) infections were risk factors for epithelial defects, while multivariate logistic regression revealed that bacterial (P = 0.039) and viral (P = 0.008) infections were more likely to result in epithelial defects after PK. Epithelial defects occurring in the setting of various causative pathogens differed significantly. Re-epithelialization depends chiefly on the regeneration of nerves [20]; studies have shown that corneal nerve density, trunk and branch numbers as observed under confocal microscopy are significantly reduced in infectious keratitis [21]. If corneal lesions involve the limbus, migration of stem cells may be adversely affected, delaying epithelial healing. Of note, eye drops containing antibiotics, glucocorticoids and various preservatives after PK have been reported to significantly impact corneal epithelial morphology and delay healing [12, 22–24]. Most of the patients in this study were from rural areas and engaged in long-term outdoor occupations. A lack of compliance with medication and hygiene also affected the healing. Epithelial defects were further aggravated by the effects of infectious keratitis on the ocular surface microenvironment after corneal transplantation, including preoperative inflammation of the meibomian glands and an uneven distribution of tears [25]. Long-term local application of drugs, systemic diseases, and aging also affect the structure and function of meibomian glands [25, 26]. Meibomian gland condition should be clinically evaluated both before and after surgery for treatment of infectious keratitis, and subsequent medical management should focus on normalizing the ocular surface microenvironment.

Corneal epithelial healing mainly relies on the recovery of corneal innervation and the integrity of limbal stem cells. Medical management, contact lenses and surgical intervention are the mainstays of treatment. In this study, 9 patients underwent suture adjustment and ultimately healed. Studies have shown that abnormal sutures affect epithelial healing by influencing the tear film stability and the movement of epithelial cells [12]. During the follow-up period of our study, 85 (74.6%) patients underwent tarsorrhaphy, and 63 eyes (74.1%) eventually healed. Although tarsorrhaphy affects appearance, it is a safe and effective method to treat serious epithelial lesions after corneal transplantation [27].Prior studies have reported that simultaneous AMT in the setting of high-risk corneal transplantation can prevent epithelial non-healing and improve graft survival [28]. For infectious keratitis patients with pertinent risk factors, appropriate adjustment of systemic and local medication in combination with AMT or tarsorrhaphy allows surgeons to tailor relatively personalized treatment and ensure best patient outcomes.

## Conclusions

We found that male sex, increased age, graft diameter >9 mm, bacterial and viral keratitis and systemic diseases (including rheumatism and cancer treated with chemo- or radiotherapy) were independent risk factors for postoperative epithelial defects. Changes in meibomian glands and the ocular microenvironment warrant greater focus from clinicians as postoperative patients with the above risk factors require appropriate early intervention in order to improve corneal graft transparency and long-term survival rate.

## Supporting information

S1 Dataset Dataset for the study. Clinical data of all patients.
(XLSX)

## References

[1] Halberstadt M, Machens M, Gahlenbek KA, Bohnke M, Garweg JG. The outcome of corneal grafting in patients with stromal keratitis of herpetic and non-herpetic origin. Br J Ophthalmol. 2002;86(6):646–52. 12034687 10.1136/bjo.86.6.646 PMC1771166

[2] Beyer CF, Byrd TJ, Hill JM, Kaufman HE. Herpes simplex virus and persistent epithelial defects after penetrating keratoplasty. AM J OPHTHALMOL. 1990;109(1):95–6. 2153344 10.1016/s0002-9394(14)75590-4

[3] Ricci F, Missiroli F, Ciotti M, Perno CF, Cerulli L. Persistent epithelial defects after penetrating keratoplasty caused by adenoviral infectious keratitis. NEW MICROBIOL. 2010;33(2):171–4. 20518280

[4] Song X, Xie L, Tan X, Wang Z, Yang Y, Yuan Y, et al A multi-center, cross-sectional study on the burden of infectious keratitis in China. PLOS ONE. 2014;9(12):e113843 https://doi:10.1371/journal.pone.0113843 25438169 10.1371/journal.pone.0113843 PMC4250054

[5] Ramamurthy S, Reddy JC, Vaddavalli PK, Ali MH, Garg P. Outcomes of Repeat Keratoplasty for Failed Therapeutic Keratoplasty. AM J OPHTHALMOL. 2016;162:83–8.https://doi:10.1016/j.ajo.2015.11.004 26558523 10.1016/j.ajo.2015.11.004

[6] Dong X, Xie L, Zhang X, et al A report on investigation and clinical application of corneal storage media[J]. Zhonghua Yan Ke Za Zhi,2000,36(1):21–23, 2. 11853576

[7] Wang T, Li S, Gao H, Shi W. Therapeutic dilemma in fungal keratitis: administration of steroids for immune rejection early after keratoplasty. Graefes Arch Clin Exp Ophthalmol. 2016;254(8):1585–9. https://doi:10.1007/s00417-016-3412-0 27342585 10.1007/s00417-016-3412-0

[8] Machado RA, Mannis MJ, Mandel HA, Feiz V, Schwab IR, Wang W, et al The relationship between first postoperative day epithelial status and eventual health of the ocular surface in penetrating keratoplasty. CORNEA. 2002;21(6):574–7. 12131033 10.1097/00003226-200208000-00008

[9] Wagoner MD, Ba-Abbad R, Al-Mohaimeed M, Al-Swailem S, Zimmerman MB. Postoperative complications after primary adult optical penetrating keratoplasty: prevalence and impact on graft survival. CORNEA. 2009;28(4):385–94. https://doi:10.1097/ICO.0b013e31818d3aef 19411956 10.1097/ICO.0b013e31818d3aef

[10] Kitzmann AS, Goins KM, Sutphin JE, Wagoner MD. Keratoplasty for treatment of Acanthamoeba keratitis. OPHTHALMOLOGY. 2009;116(5):864–9. https://doi:10.1016/j.ophtha.2008.12.029 19410943 10.1016/j.ophtha.2008.12.029

[11] Feiz V, Mannis MJ, Kandavel G, McCarthy M, Izquierdo L, Eckert M, et al Surface keratopathy after penetrating keratoplasty. Trans Am Ophthalmol Soc. 2001;99:159–68, 168–70. 11797303 PMC1359006

[12] Shimazaki J, Shimmura S, Mochizuki K, Tsubota K. Morphology and barrier function of the corneal epithelial after penetrating keratoplasty: association with original diseases, tear function, and suture removal. CORNEA. 1999;18(5):559–64. 10487430

[13] Mashaghi A, Hong J, Chauhan SK, Dana R. Ageing and ocular surface immunity. Br J Ophthalmol. 2017;101(1):1–5. https://doi:10.1136/bjophthalmol-2015-307848 27378485 10.1136/bjophthalmol-2016-EGSguideline.001 PMC5583682

[14] Chou L, Cohen EJ, Laibson PR, Rapuano CJ. Factors associated with epithelial defects after penetrating keratoplasty. Ophthalmic Surg. 1994;25(10):700–3. 7898864

[15] Kamble N, Sharma N, Maharana PK, Bandivadekar P, Nagpal R, Agarwal T, et al Evaluation of the Role of Umbilical Cord Serum and Autologous Serum Therapy in Reepithelialization After Keratoplasty: A Randomized Controlled Clinical Trial. EYE CONTACT LENS. 2017;43(5):324–9. https://doi:10.1097/ICL.0000000000000277 27196995 10.1097/ICL.0000000000000277

[16] Fu Y, Liu J, Tseng SC. Ocular surface deficits contributing to persistent epithelial defects after penetrating keratoplasty. CORNEA. 2012;31(7):723–9. https://doi:10.1097/ICO.0b013e31821142ee 22495035 10.1097/ICO.0b013e31821142ee

[17] Kawamoto K, Morishige N, Yamada N, Chikama T, Nishida T. Delayed corneal epithelial wound healing after penetrating keratoplasty in individuals with lattice corneal dystrophy. AM J OPHTHALMOL. 2006;142(1):173–4. https://doi:10.1016/j.ajo.2006.01.077 16815275 10.1016/j.ajo.2006.01.077

[18] Mannis MJ, Zadnik K, Miller MR, Marquez M. Preoperative risk factors for surface disease after penetrating keratoplasty. CORNEA. 1997;16(1):7–11. 8985626

[19] Dan J, Zhou Q, Zhai H, Cheng J, Wan L, Ge C, et al Clinical analysis of fungal keratitis in patients with and without diabetes. PLOS ONE. 2018;13(5):e196741 https://doi:10.1371/journal.pone.0196741 29715322 10.1371/journal.pone.0196741 PMC5929555

[20] Tsubota K, Mashima Y, Murata H, Yamada M, Sato N. Corneal epithelial following penetrating keratoplasty. Br J Ophthalmol. 1995;79(3):257–60. 7703205 10.1136/bjo.79.3.257 PMC505074

[21] Cruzat A, Witkin D, Baniasadi N, Zheng L, Ciolino JB, Jurkunas UV, et al Inflammation and the nervous system: the connection in the cornea in patients with infectious keratitis. Invest Ophthalmol Vis Sci. 2011;52(8):5136–43. https://doi:10.1167/iovs.10-7048 21460259 10.1167/iovs.10-7048 PMC3176064

[22] Kimakura M, Usui T, Yokoo S, Nakagawa S, Yamagami S, Amano S. Toxicity of topical antifungal agents to stratified human cultivated corneal epithelial sheets. J Ocul Pharmacol Ther. 2014;30(10):810–4. https://doi:10.1089/jop.2014.0044 25280055 10.1089/jop.2014.0044 PMC4259201

[23] Sugar A, Bokosky JE, Meyer RF. A randomized trial of topical corticosteroids in epithelial healing after keratoplasty. CORNEA. 1984;3(4):268–71. 6400576

[24] Moshirfar M, Marx DP, Kumar R. The effect of the fourth-generation fluoroquinolones on corneal reepithelialization after penetrating keratoplasty. CORNEA. 2005;24(7):833–6. 16160500 10.1097/01.ico.0000157420.11448.d4

[25] Mizoguchi S, Iwanishi H, Arita R, Shirai K, Sumioka T, Kokado M, et al Ocular surface inflammation impairs structure and function of meibomian gland. EXP EYE RES. 2017;163:78–84. https://doi:10.1016/j.exer.2017.06.011 28950941 10.1016/j.exer.2017.06.011 PMC5914509

[26] Schaumberg DA, Nichols JJ, Papas EB, Tong L, Uchino M, Nichols KK. The international workshop on meibomian gland dysfunction: report of the subcommittee on the epidemiology of, and associated risk factors for, MGD. Invest Ophthalmol Vis Sci. 2011;52(4):1994–2005. https://doi:10.1167/iovs.10-6997e 21450917 10.1167/iovs.10-6997e PMC3072161

[27] Cosar CB, Cohen EJ, Rapuano CJ, Maus M, Penne RP, Flanagan JC, et al Tarsorrhaphy: clinical experience from a cornea practice. CORNEA. 2001;20(8):787–91. 11685052 10.1097/00003226-200111000-00002

[28] Seitz B, Das S, Sauer R, Hofmann-Rummelt C, Beckmann MW, Kruse FE. Simultaneous amniotic membrane patch in high-risk keratoplasty. CORNEA. 2011;30(3):269–72. https://doi:10.1097/ICO.0b013e3181eae8ea 21304288 10.1097/ICO.0b013e3181eae8ea

